# Causal relationship between insomnia and tuberculosis: A bi-directional Mendelian randomization analysis

**DOI:** 10.1097/MD.0000000000030509

**Published:** 2022-09-16

**Authors:** Shaobin Zhang, Wei Zhang, Yan Feng, Shiqian Wan, Jing Ge, Zhaohui Qu, Xin Li

**Affiliations:** a Department of Surgery, Wuhan Jinyintan Hospital, Tongji Medical College of Huazhong University of Science and Technology, Wuhan, China; b Department of Critical Care Medicine, Wuhan Jinyintan Hospital, Tongji Medical College of Huazhong University of Science and Technology, Wuhan, China; c Department of Tuberculosis, Wuhan Jinyintan Hospital, Tongji Medical College of Huazhong University of Science and Technology, Wuhan, China; d Department of Infectious Diseases, Wuhan Jinyintan Hospital, Tongji Medical College of Huazhong University of Science and Technology, Wuhan, China.

**Keywords:** insomnia, instrumental variant, Mendelian randomization, single-nucleotide polymorphism, tuberculosis

## Abstract

Previous observational studies appear to have established a bi-directional association between sleep disorders and tuberculosis. However, their conclusions are prone to be biased by confounding effects and reverse causation due to the nature of observational studies. Mendelian randomization (MR) approach provides unconfounded estimates of causal effects and overcomes the limitations of observational studies. We performed a bi-directional MR analysis to clarify whether there existed a causal effect of insomnia on tuberculosis, or tuberculosis on insomnia. In forward-direction MR, we chose genome-wide significant (*P* < .5 × 10^–8^) and independent (*r*^2^ < 0.001) single-nucleotide polymorphisms (SNPs) as instrumental variants (IVs), then extracted effect estimates of these IVs in tuberculosis genome-wide association study (GWAS) dataset to explore causal effect of genetically proxied insomnia on tuberculosis using inverse variance-weighted (IVW), MR-Egger, and weighted median methods. Additionally, we examined robustness and pleiotropy of effect estimates by heterogeneity and sensitivity analysis. Similarly, we investigated causal effect of genetically proxied tuberculosis on insomnia in reverse-direction MR. We revealed no causal relationship between genetically proxied insomnia and tuberculosis using 15 SNPs in forward-direction MR (IVW OR 5.305 [0.100–281.341], *P* = .410) and reverse-direction MR analysis (ORs and *P* values were not applicable due to no eligible SNPs in GWAS), with insignificant heterogeneity (*Q* = 22.6, *I*^2^ < 0.001, *P* = .066) and pleiotropy (intercept = 0.032, SE = 0.057, *P* = .592) in effect estimates. Our bi-directional MR analysis affirms no causal effect of insomnia on tuberculosis, or tuberculosis on insomnia.

## 1. Introduction

As a modifiable lifestyle behavior, sleep is vital to human health.^[1]^ Previous observational studies have documented that sleep disorders predispose people to tuberculosis^[2–4]^; on the other hand, the patients suffering from tuberculosis are often faced with sleep disorders,^[5]^ suggesting an association between sleep disorders and tuberculosis. It is worth noting that the conclusions from observational studies are prone to be biased by confounding effects and reverse causation,^[1,6,7]^ even when they are well designed, or prospective with large sample sizes.

Mendelian randomization (MR) is a new approach using genetic variants that are robustly associated with potentially modifiable risk factors as instrumental variants (IVs) to examine the causal effect of exposure on outcome,^[8–10]^ and to establish a theoretical basis for further prevention.^[6]^ The rationale for the MR design is that the genetic variants are randomly assigned from parents to offspring at conception and are unchanged through a lifetime.^[1,6,11]^ Thus, the MR design can be conceptualized as a natural experiment^[1]^ and is less susceptible to the effects of confounding and reverse causation bias that are often encountered in observational studies.^[1,6,11,12]^ Accordingly, MR provides unconfounded estimates and overcomes the limitations of observational studies,^[6,13]^ given 3 key assumptions are satisfied: IVs must be robustly associated with the exposure in question; IVs must not be linked in any way to confounding variables in the relationship between genetic variants and the outcome of interest; and IVs merely affect the outcome of interest via their association with the exposure of interest, with no alternative pathways coming into play.^[14–16]^ Genetic variants robustly associated with insomnia have recently been identified in large genome-wide association studies (GWASs) with sample sizes of around 50,000 to more than 1 million.^[17–25]^ Findings from these GWASs have verified the impact of several core genes on insomnia and identified genetic variants playing a circadian role that have not been previously known.^[26]^

However, to our knowledge, there has been no study attempting to employ MR method to clarify whether there exists a causal effect of sleep disorders on risk of tuberculosis occurrence, or reversely, a causal effect of tuberculosis on susceptibility to insomnia yet. Therefore, the causality behind such a bi-directional association remains largely ambiguous. In response, we designed and fulfilled a bi-directional MR analysis to explore the potential causal relationship between insomnia, one ingredient of sleep disorders, and tuberculosis.

## 2. Materials and Methods

### 2.1. Study design

We designed a bi-directional MR study in the principles of 2-sample MR to: figure out whether genetically proxied insomnia (i.e., single-nucleotide polymorphisms [SNPs] for exposure) causally affected tuberculosis risk (i.e., SNPs for outcome) in forward-direction MR analysis; and make clear whether genetic predisposition to tuberculosis (i.e., SNP for exposure) causally affected insomnia risk (i.e., SNPs for outcome) in reverse-direction MR analysis.

### 2.2. Data source

We chose SNPs as IVs for insomnia (The Medical Research Council Integrative Epidemiology Unit at the University of Bristol Consortium; GWAS ID: ukb-b-3957; n = 462,341; https://gwas.mrcieu.ac.uk/datasets/ukb-b-3957/), and for tuberculosis (Tuberculosis Modelling and Analysis Consortium; GWAS ID: finn-a-tuberculosis; n = 96,499; https://gwas.mrcieu.ac.uk/datasets/finn-a-tuberculosis/) to conduct MR analyses using publicly available summary statistics deposited in the IEU GWAS database (https://gwas.mrcieu.ac.uk/).^[27]^ To avoid population bias, we selected SNPs and their corresponding summary statistics (*P* value, *β* effect, and standard error [SE]) from the studies enrolling only individuals of European ancestry for both insomnia and tuberculosis. Ethical review and informed consent were obtained from the original GWASs.

### 2.3. Selection of instrumental variables

We implemented a series of quality control steps to choose eligible IVs. Specifically, the SNPs associated with insomnia at genome-wide significance (*P* < 5 × 10^–8^) in “ukb-b-3957” dataset were selected as IVs for insomnia.^[28]^ We clumped SNPs to achieve independent loci with a threshold of linkage disequilibrium (LD) *r^2^* = 0.001 and distance of 10,000 kb.^[29]^ Then, we extracted the effect estimates of the selected IVs from “finn-a-tuberculosis” dataset. The SNPs with a minor allele frequency (MAF) of <0.01 were excluded.^[30]^ Also, we underwent a reverse-direction MR analysis in order to investigate a potential causal effect of a genetically proxied tuberculosis on insomnia risk. To that end, we likewise selected SNPs that were genome-wide significant (*P* < 5 × 10^–8^) and independently inherited (*r^2^* < 0.001) without LD for tuberculosis from “finn-a-tuberculosis” dataset, and then extracted the corresponding effect estimates of the selected IVs from “ukb-b-3957” dataset. These rigorously selected SNPs were used as the final instruments for the MR analyses.

### 2.4. Strength of SNPs in explaining phenotypic variation

First, we computed *r*^2^-value, the proportion of phenotypic variation explained by each SNP, using the formula: *r*^2 ^= 2 × (1 – MAF) × MAF × *β*^2^/(SE^2^ × N) where SE (*β*) is the SE (*β* coefficient) for effect size, MAF is the minimum allele frequency for each SNP, and N is the sample size. Then, in order to satisfy the first MR assumption, we calculated an *F*-statistic to evaluate the total strength of the 15 selected SNPs in explaining phenotypic variation by using the formula: [(N – *k* – 1)/*k*] × [*r*^2’^/(1 – *r*^2’^)] where N is the sample size, *k* is the total number of SNPs selected for MR analysis, and *r*^2’^ is the sum of *r*^2^ values for all the 15 SNPs. An *F*-statistic > 10 suggests that the full set of instrumental SNPs are sufficiently strong to lessen any potential bias,^[31]^ while an *F*-statistic ≤ 10 implies “weak instruments.”

### 2.5. Removal of confounding and palindromic SNPs

In the context of insomnia-tuberculosis relationship, such tuberculosis-relevant traits as body weight,^[32,33]^ body mass index,^[32,33]^ diabetes mellitus (DM),^[34,35]^ alcohol intake,^[36]^ and smoking^[36,37]^ are most likely to be potential and major confounders. To address the second MR assumption, we inquired for each IV and its proxied traits referring to PhenoScannerV2 database (http://www.phenoscanner.medschl.cam.ac.uk/) and removed the IVs arrogating these tuberculosis-relevant traits at a threshold of *r^2^* > 0.80.^[38,39]^ We harmonized the insomnia and tuberculosis data by removing all palindromic SNPs with intermediate allele frequencies out of the selected instrumental SNPs above,^[40]^ where palindromic SNPs referred to the SNPs whose alleles correspond to nucleotides that pair with each other in a DNA molecule, and intermediate allele frequencies denoted the allele frequencies between 0.01 and 0.30.^[30]^

### 2.6. Estimation of causal effect

After we determined the list of SNPs according to the selection criteria above, we executed forward MR analysis to estimate the overall effects of the selected SNPs for insomnia on tuberculosis using inverse variance-weighted (IVW), MR-Egger, and the weighted median (WM) methods.^[41]^ Given that the results could be biased by the horizontal pleiotropy of IVs, we validated the stability of the results by comparing the effect estimates across the 3 MR methods. The IVW method uses a meta-analysis approach to combine the Wald ratios of the causal effects of each SNP, assuming all SNPs are valid IVs with no evidence of directional pleiotropy, we considered that the IVW method provides the most precise estimates.^[30]^ Thereafter, we applied the same MR methods as above in reverse-direction MR analysis. Effect estimates were reported in odds ratio (OR) with 95% confidence interval (CI).

### 2.7. Heterogeneity and sensitivity analysis

To process the third MR assumption, we conducted heterogeneity assessment and sensitivity analyses to verify whether heterogeneity and pleiotropy within the genetic instruments biased the MR results. Cochran *Q*-statistics and *I*^2^-values were used to quantitatively estimate the level of heterogeneity between SNPs.^[42]^ “Leave-one-out” sensitivity analysis was performed by removing a different SNP in each iteration to estimate the causal effect of outlying SNPs, and to guarantee that the MR estimates were not affected by removing SNPs.^[43]^ In order to ascertain the presence of pleiotropy, and to provide outlier-adjusted estimates by removing any pleiotropic outlying SNPs, we additionally applied the Pleiotropy RESidual Sum and Outlier (PRESSO) analysis.^[44]^

### 2.8. Statistical analysis

**Figure 1.**
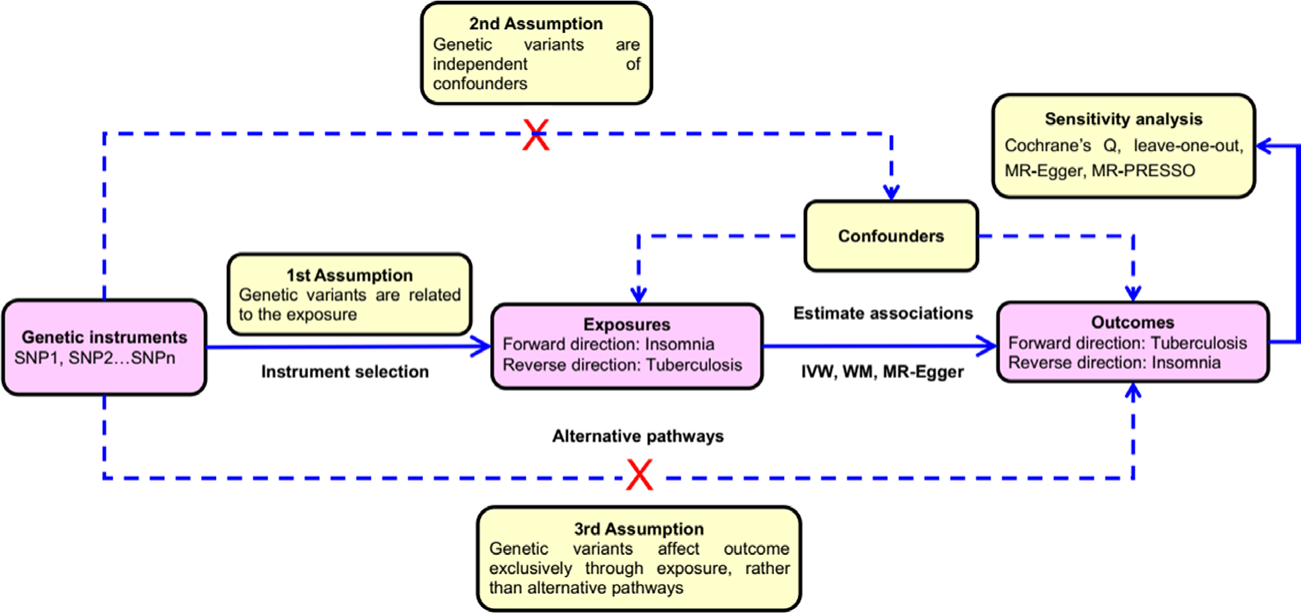
Paradigm and schematic model of bi-directional MR analysis. Three key MR assumptions: (1) genetic variants are associated with exposures (i.e., valid instruments); (2) genetic variants are not associated with any confounder of the exposure-outcome association; (3) genetic variants only affect outcomes via its association with exposures (i.e., no horizontal pleiotropy). IVW = inverse variance-weighted, MR = Mendelian randomization, PRESSO = Pleiotropy RESidual Sum and Outlier, SNP = single-nucleotide polymorphism, WM = weighted median.

All statistical analyses were conducted using R statistical software with the “devtools,” “TwoSampleMR,” “LDlinkR,” and “MRPRESSO” Packages (version 4.1.0, R Foundation for Statistical Computing, Vienna, Austria). All statistical tests were 2-sided, and the results of the MR analyses and sensitivity analyses regarding the causal effects of exposures and outcomes were considered statistically significant at *P* < .05. The rationale and procedures for our bi-directional MR study are elucidated in Figure 1.

## 3. Results

### 3.1. Forward-direction MR analysis

#### 3.1.1. SNP screening.

To explore the causal effect of insomnia on tuberculosis risk, 42 genome-wide significant (*P* < 5 × 10^–8^) and independently inherited (*r^2^* < 0.001) SNPs without LD were preliminarily included as IVs for insomnia. Out of the 42 SNPs, 10 SNPs associated with at least 1 of tuberculosis-relevant traits such as body weight, body mass index, DM, alcohol intake, and smoking, were excluded by inquiring the PhenoScanner V2 database; 17 SNPs were removed because their MAF < 1%. None of the candidate SNPs was detected to be palindromic. Finally, a total of 15 SNPs (rs11097861, rs113851554, rs11790060, rs17151854, rs17709610, rs2604551, rs4572538, rs4577309, rs56093896, rs6975972, rs72924721, rs7711696, rs931221, rs9570080, and rs9845387) were accepted for MR analyses of causal effect of insomnia on tuberculosis risk, as exhibited in Table 1. The association between each SNP and insomnia, and that between each SNP and tuberculosis are also presented in Table 1.

**Table 1:** Characteristics of SNPs used in forward MR analysis for causal effect of insomnia on tuberculosis risk.

Target SNPs*	Chr	Effect allele (alternative)	Association with insomnia				MAF	*r*^2^ †	Association with tuberculosis			
			*β*	SE	EAF	*P*			*β*	SE	EAF	*P*
rs11097861	4	G (A)	0.0100	0.0016	0.7163	1.10 × 10^−9^	0.3182	0.00003480	–0.0335	0.0731	0.6905	.6464
rs113851554	2	T (G)	0.0468	0.0033	0.0573	2.90 × 10^−45^	0.0455	0.00003745	0.0060	0.1292	0.0733	.9629
rs11790060	9	C (T)	–0.0103	0.0016	0.3308	5.80 × 10^−11^	0.3283	0.00004092	0.1134	0.0700	0.3570	.1053
rs17151854	8	T (G)	0.0130	0.0021	0.1524	3.80 × 10^−10^	0.1970	0.00002683	0.0480	0.1011	0.1271	.6348
rs17709610	10	G (A)	–0.0099	0.0016	0.2980	9.50 × 10^−10^	0.3232	0.00003542	–0.0325	0.0753	0.2748	.6663
rs2604551	4	G (T)	–0.0085	0.0016	0.6404	4.70 × 10^−8^	0.2879	0.00002647	–0.2412	0.0761	0.7287	.0015
rs4572538	2	T (C)	–0.0096	0.0016	0.3641	7.70 × 10^−10^	0.4343	0.00004021	–0.0249	0.0687	0.4060	.7172
rs4577309	2	G (A)	–0.0085	0.0015	0.5337	1.00 × 10^−8^	0.4747	0.00003540	–0.0220	0.0681	0.4323	.7463
rs56093896	2	A (C)	–0.0124	0.0018	0.2141	7.70 × 10^−12^	0.2121	0.00003386	–0.0477	0.0820	0.2138	.5606
rs6975972	7	G (A)	–0.0090	0.0015	0.5787	2.00 × 10^−9^	0.4242	0.00003800	–0.0871	0.0674	0.5551	.1964
rs72924721	11	T (C)	0.0165	0.0029	0.0731	1.10 × 10^−8^	0.0657	0.00000869	–0.1028	0.1207	0.0858	.3942
rs7711696	5	T (G)	0.0112	0.0016	0.3050	4.10 × 10^−12^	0.3384	0.00004655	–0.1038	0.0727	0.3017	.1536
rs931221	12	A (T)	0.0106	0.0018	0.2367	1.30 × 10^−9^	0.2778	0.00003194	0.0817	0.0931	0.1574	.3803
rs9570080	13	C (T)	–0.0106	0.0016	0.3441	1.60 × 10^−11^	0.3434	0.00004429	–0.0232	0.0677	0.4447	.7314
rs9845387	3	A (C)	–0.0219	0.0038	0.0403	7.10 × 10^−9^	0.0253	0.00000357	–0.3304	0.1548	0.0507	.0328

Chr = chromosome, EAF = effect allele frequency, MAF = minor allele frequency, MR = Mendelian randomization, SE = standard error, SNP = single nucleotide polymorphism, *β* = regression effect size.

* Ten SNPs associated with confounding traits of tuberculosis (rs314280, rs705219, rs1430205, rs2014830, rs2297787, rs2644128, rs2803296, rs10838708, rs11635495, and rs56365214), and 17 SNPs with MAF < 1% (rs11152363, rs12049261, rs12470989, rs1547630, rs1592757, rs1988337, rs2062113, rs224032, rs324017, rs4886860, rs56330606, rs6561715, rs6690017, rs68094047, rs8180817, rs9894577, and rs9906181) are excluded. None of candidate SNP is found to be palindromic. Eventually, a total of 15 SNPs is eligible for MR analyses.

† r^2^ denotes proportion of phenotypic variation explained by each SNP.

#### 3.1.2. Strength of SNPs in explaining phenotypic variation.

The 15 selected SNPs together accounted for approximately 0.048% (summary *r*^2^ value) of the phenotypic variation in insomnia (Table 1). For these SNPs, the value of summary *F*-statistic was 14.9372, larger than 10, suggesting that the full set of 15 SNPs sufficiently met the strong relevance assumption of MR (i.e., first MR assumption), and that the instrumental bias was weak and unlikely to substantially affect the estimation of causal effect.

#### 3.1.3. MR estimates of causal effect.

**Figure 2.**
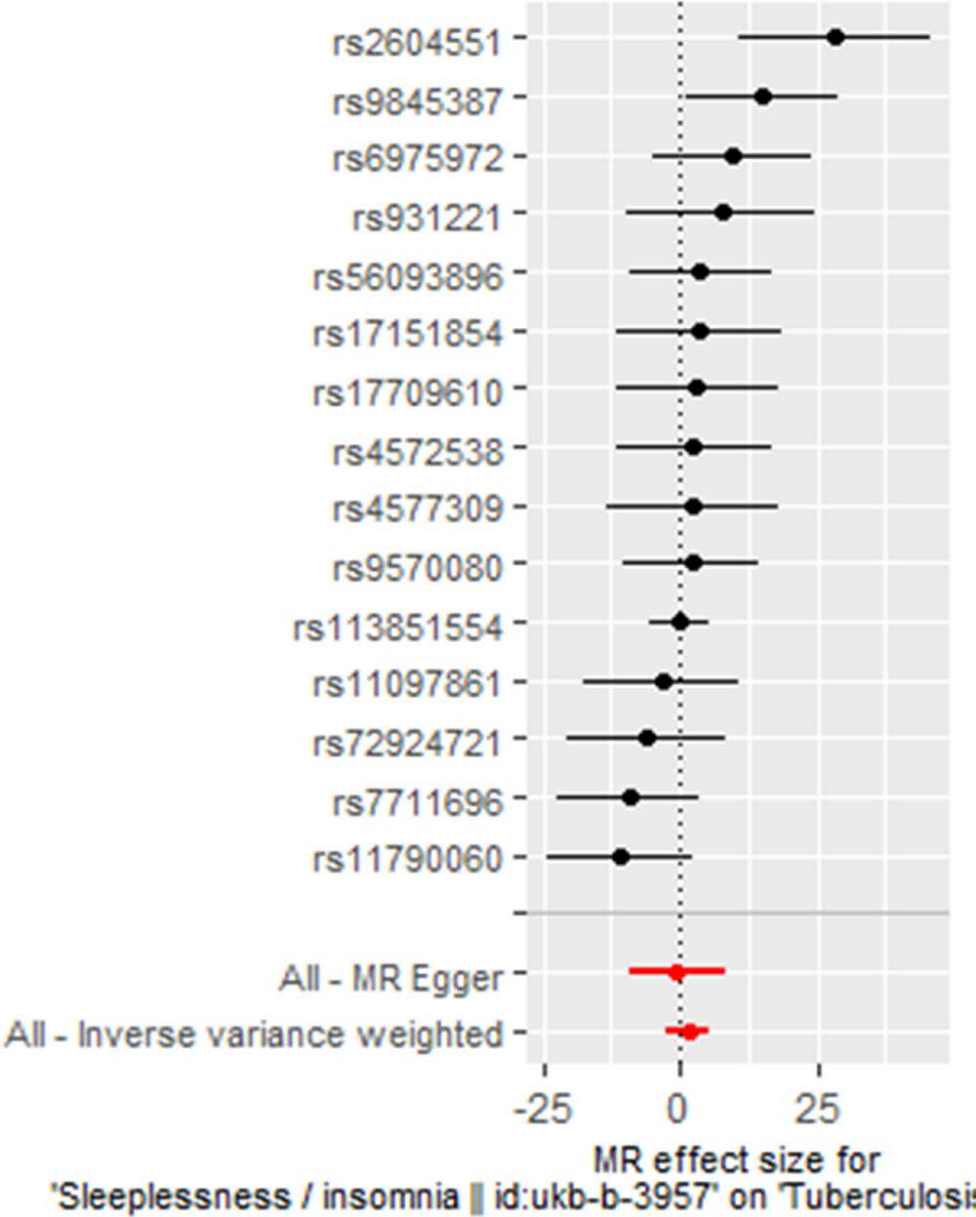
Forrest plot depicting MR estimates of causal effect of insomnia on tuberculosis risk, suggesting no causal relationship between genetically proxied insomnia and tuberculosis risk based on MR-Egger (*P* > .05) and IVW methods (*P* > .05) using full set of 15 SNPs. IVW = inverse variance-weighted, MR = Mendelian randomization, SNP = single-nucleotide polymorphism.

**Figure 3.**
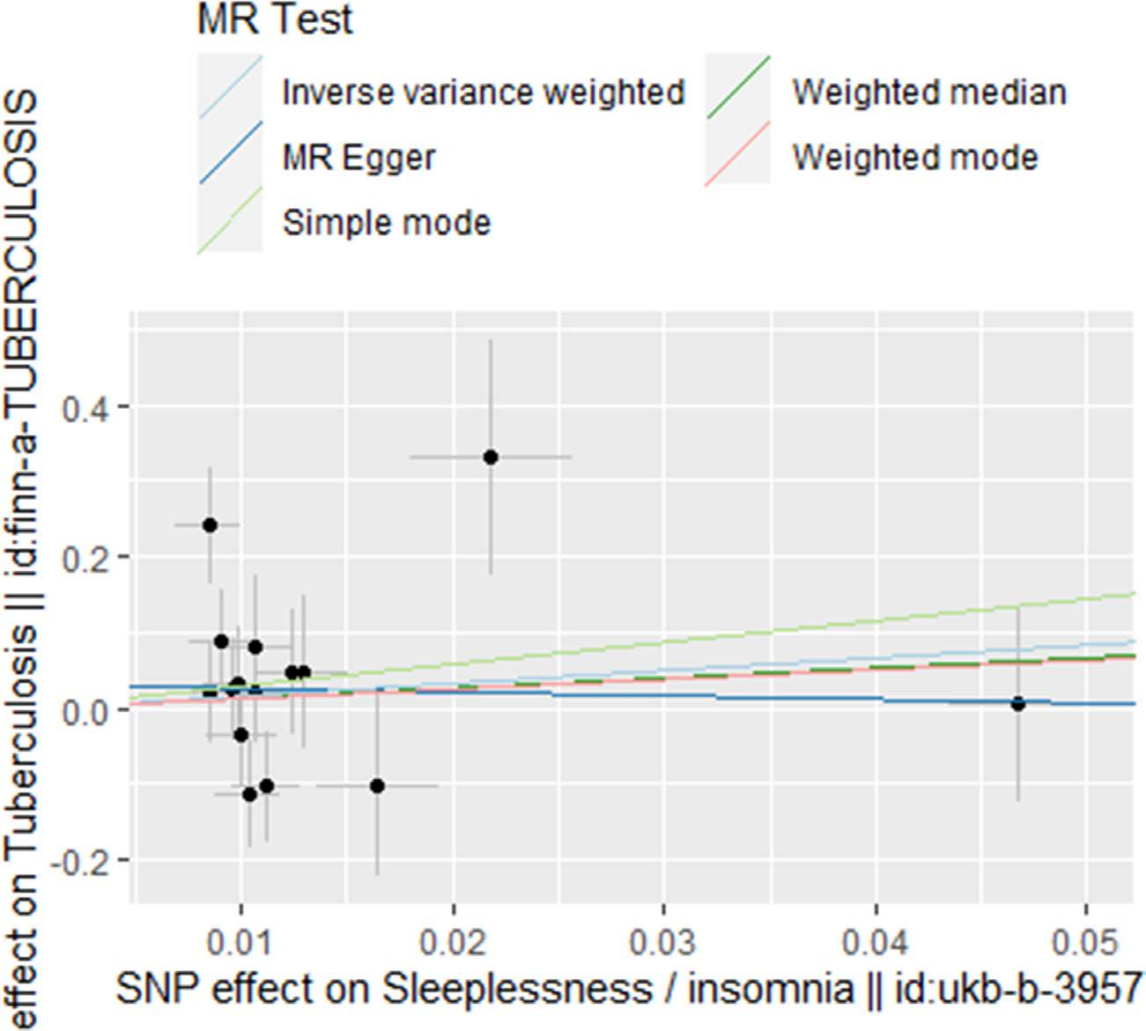
Scatter plot illustrating SNP-tuberculosis association against SNP-insomnia association using different MR methods. MR = Mendelian randomization, SNP = single-nucleotide polymorphism.

As displayed in Table 2, Figures 2, and 3, there is no evidence indicating a positive or reverse causal relationship between genetically proxied insomnia and tuberculosis risk based on the IVW, WM, and MR-Egger methods using the full set of 15 SNPs (IVW OR 5.3049 [0.1000 – 281.3405], *P* = .4102; WM OR 3.8608 [0.0325 – 458.0857], *P *= .5793; MR Egger OR 0.6301, 95% CI [0.0001 – 3491.0209], *P* = .9180). Given that the IVW estimate is consistently insignificant with the estimates yielded by the other 2 MR methods, and that IVW estimate is most likely to be unbiased and the most precise, we believe that suffering insomnia does not have any causal effect on susceptibility to tuberculosis.

**Table 2:** MR estimates for causal effect of insomnia on tuberculosis with 3 MR methods.

Method	No. of SNPs	OR (95% CI)	*β*	SE	*P*
IVW	15	5.3049 (0.1000–281.3405)	–0.4618	4.3978	.410
WM	15	3.8608 (0.0325–458.0857)	1.3509	2.4368	.579
MR Egger	15	0.6301 (0.0001–3491.0209)	1.6686	2.0260	.918

CI = confidence interval, IVW = inverse variance-weighted, MR = Mendelian randomization, OR = odds ratio, SE = standard error, SNP = single-nucleotide polymorphism, WM = weighted median.

#### 3.1.4. Heterogeneity and pleiotropy.

**Figure 4.**
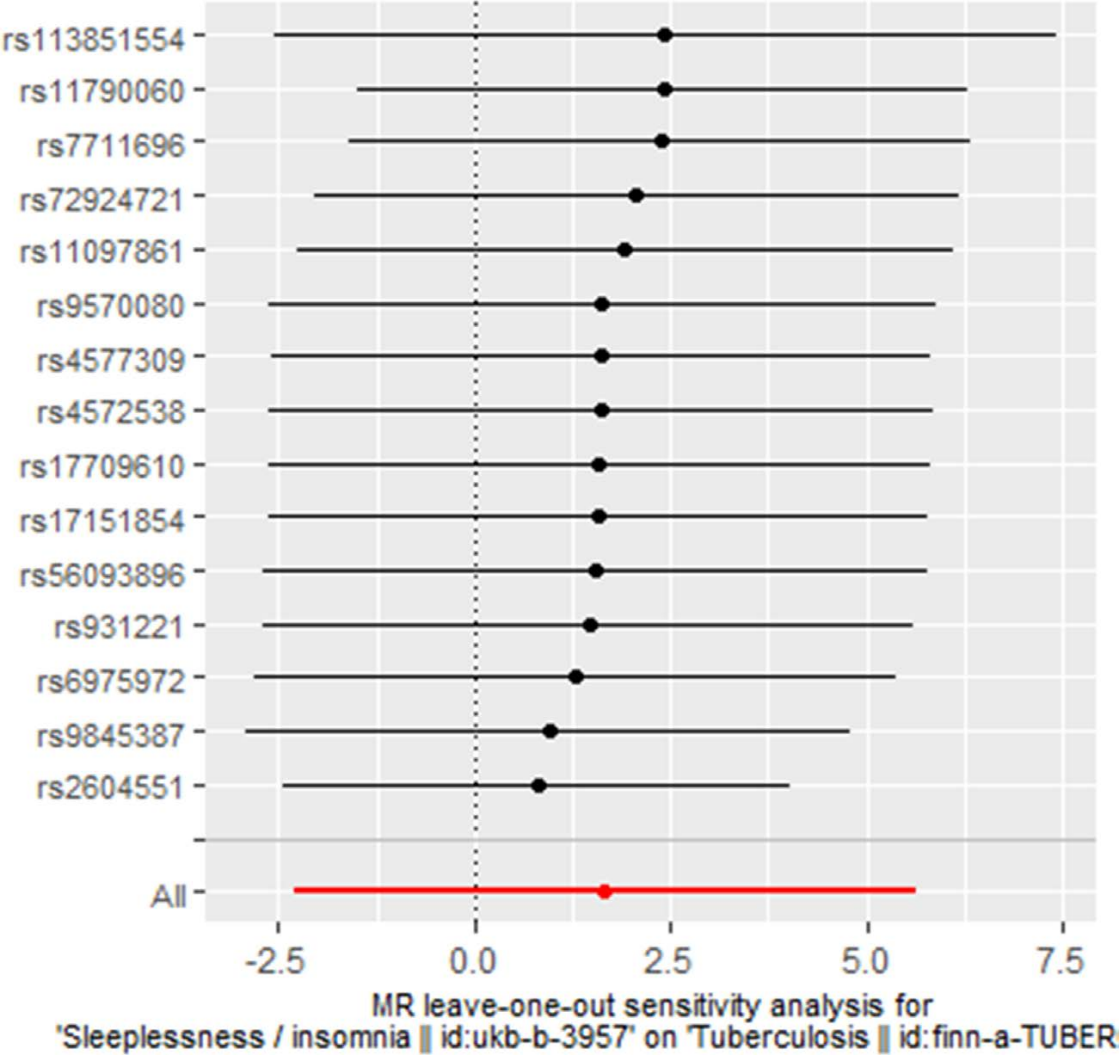
Plot for “leave-one-out” analysis of causal effect of genetically proxied insomnia on tuberculosis risk, revealing no single SNP altering MR estimates when each SNP is removed from the principal MR analysis. The red line is representative of the significance contributed by overall MR estimate using the IVW method. IVW = inverse variance-weighted, MR = Mendelian randomization, SNP = single-nucleotide polymorphism.

Regarding the estimates determined by IVW and MR-Egger methods, our *Q*-statistics and *I^2^*-values indicated no notable heterogeneity (IVW method: *Q* = 22.6, *I*^2^ < 0.001, *P* = .066; MR-Egger method: *Q* = 22.1, *I*^2^ < 0.001, *P* = .053). Then, our “leave-one-out” analysis illustrated that the MR estimates would be significantly altered when none of the 15 single SNPs was removed from the MR analyses (Fig. 4 and Table 3). Furthermore, MR-Egger method detected no pleiotropic effect in regard to the MR estimate of the causal effect of genetically proxied insomnia on tuberculosis risk (intercept = 0.0315, SE = 0.057, *P* = .592). Coincidently, the outlier-corrected MR-PRESSO method identified no outlying SNP for estimating the causal association of genetically proxied insomnia with tuberculosis risk (causal estimate = 1.669, standard deviation = 2.026, *T*-statistic = 0.824, *P* = .424). Therefore, the null causal effect in our principal MR analysis was robust and unbiased, which was evidenced by neither noticeable heterogeneity nor detectable pleiotropy. Conclusively, our principal MR analyses with 3 methods, and sensitivity analyses using IVW, MR Egger, and MR-PRESSO methods, consistently establish that there exists no causal effect of suffering insomnia on increased risk of tuberculosis occurrence.

**Table 3:** Effect estimates of 15 single SNPs for relationship between genetically determined insomnia and tuberculosis risk.

SNP	*β*	SE	*P*
rs11097861	1.920	2.131	.367
rs113851554	2.436	2.547	.339
rs11790060	2.409	1.979	.224
rs17151854	1.580	2.145	.461
rs17709610	1.595	2.148	.458
rs2604551	0.798	1.647	.628
rs4572538	1.620	2.156	.452
rs4577309	1.631	2.145	.447
rs56093896	1.534	2.161	.478
rs6975972	1.288	2.094	.538
rs72924721	2.061	2.095	.325
rs7711696	2.367	2.019	.241
rs931221	1.463	2.115	.489
rs9570080	1.634	2.171	.452
rs9845387	0.952	1.969	.629
All	1.669	2.026	.410

SE = standard error, SNP = single-nucleotide polymorphism.

### 3.2. Reverse-direction MR analysis

Unfortunately, in our reverse-direction MR analysis, no genome-wide significant (*P* < 5 × 10^–8^) and independently inherited (*r*^2^ < 0.001) SNP was appropriate for proxy of tuberculosis to investigate the causal effect of suffering tuberculosis on insomnia risk. Affirmatively, there exists no causal effect of genetically proxied tuberculosis on risk of insomnia occurrence.

## 4. Discussion

### 4.1. Main findings

Using a combination of 15 SNPs, which were validated to be as a strong genetic instrument for explaining phenotypic variation regarding insomnia, we conducted a forward directional MR analysis and evidenced no causal effect of suffering insomnia on tuberculosis predisposition. Moreover, our result of null causal effect in our principal MR analysis (i.e., IVW-MR method) was robust, unbiased, and reliable, in consideration of neither noticeable heterogeneity nor detectable pleiotropy. Besides, our result of reverse directional MR analysis expelled the reverse causal effect of suffering tuberculosis on insomnia risk.

### 4.2. Sleep disorders on tuberculosis

There have been several observational studies investigating the associations between sleep disorders and the predisposition of people to tuberculosis. Pelders et al^[2]^ undertook a cross-sectional study among the workers in the South African mining industry, informing that sleep was among socio-demographic factors associated with tuberculosis. Kou et al^[3]^ conducted a case-control study with multivariate logistic regression analysis to explore the relationship between sleep quality and the risk of active pulmonary tuberculosis (PTB) in Shandong Province, China, and drew a conclusion that poor sleep quality was an independent risk factor for PTB among DM patients with a course of > 5 years, pinpointing an imperative epidemiological implication of sleep quality for PTB control. Additionally, inadequate sleep duration has been identified as a risk factor for an approximately 3-fold higher odds of multidrug-resistant tuberculosis (Adjusted OR: 2.77, 95% CI: 1.11–6.92, *P* < .05) in a nationwide case-control study with multivariate logistic regression analysis undertaken by Tenzin et al.^[4]^ However, the conclusions that were drawn from these observational studies were based on heterogenous types of study design (cross-sectional study^[2]^ or case-control study^[3,4]^), participants (general subjects^[2,4]^ or DM patients^[3]^), and definitions for exposure events (sleep quality^[2,3]^ or sleep duration^[4]^) and outcome events (tuberculosis,^[2]^ PTB,^[3]^ or multidrug-resistant tuberculosis). The diversity existing these observational studies compromised the homogeneity, reliability, and generalizability of their conclusions and hindered us from synthesizing consistent evidence. Moreover, these results acquired from previous observational studies were not in line with our MR findings in the forward direction that we did not recognize any causal effect of suffering insomnia on susceptibility to tuberculosis in individuals of European descent based on the IVW, WM, and MR-Egger methods using the full set of 15 SNPs. Our MR analyses, for the first time, resolved the ambiguity deposited in previous observational studies, and definitely denied any genetically causal effect of suffering insomnia on tuberculosis risk. The divergence between the results from previous observational studies and that acquired from our MR analyses can be interpreted by, as we have described in the Section of Introduction, potential methodological limitations in observational studies and inherent superiority of MR analyses over observational studies since MR analyses can overcome the issues of confounding and reverse causality by integrating a set of SNPs that are strongly associated with an exposure trait and estimate causal effect of insomnia on one outcome event, that is, tuberculosis risk.

### 4.3. Tuberculosis on sleep disorders

On the other hand, in a cross-sectional study performed by Raj et al,^[5]^ poor-quality sleep was more prevalent in pulmonary or extrapulmonary TB patients than in normal population in Bengaluru, India. Inconsistent with Raj et al’s study, our MR analyses in reverse direction did not come up with a causal association of tuberculosis with increased risk of insomnia in the same population using the same set of 15 SNPs. The discrepancies concerning findings between our MR analyses and previous observational studies are possibly interpreted by adjustment for various confounders that underlie the relationships between sleep disorders and tuberculosis risk. Our MR analyses show a superiority over these observational studies in terms of considerably reduced confounding bias, since some demographic confounders, such as age and sex, have been well adjusted in the corresponding original GWAS, and 10 SNPs associated with confounding traits of tuberculosis have been excluded referring to PhenoScannerV2 database.

### 4.4. Comparisons across 3 MR methods

Given that the IVW estimates are consistent with the WM estimates and that the IVW estimates may be unbiased and are considerably more powerful than the MR-Egger estimates,^[30]^ we strengthen our advocate of null causal effect of insomnia on tuberculosis.

### 4.5. Heterogeneity and pleiotropy

Moreover, multiple heterogeneity and sensitivity analyses have been conducted to detect and remove any potential pleiotropy, reassuring that our MR estimates are robust and reliable, with no perceptible bias from other sources of pleiotropy.

### 4.6. Implications

With minimized confounding and reverse causation bias, our bi-directional MR analysis provides an evidence of no causal effect of insomnia on tuberculosis, or tuberculosis on insomnia. In other words, neither of these 2 biological traits is a consequence nor the cause of each other, which suggests that insomnia and tuberculosis might not share a common pathogenesis. Our findings potentially have some implications for public health, in that they may serve as an addition to insomnia and tuberculosis research, and that they bring some new information with respect to the assumed disparities in the pathogenesis of the 2 diseases.

### 4.7. Strength

To the best of our knowledge, our study is the first MR study focused on bi-directional causal relationship between insomnia and tuberculosis using large-scale GWAS data. The strengths of this study include the large sample size from GWAS summary datasets, appreciably lessened confounding and reverse causation bias compared to observational studies, and good robustness without apparent heterogeneity or pleiotropy in findings in regard to the estimate of causal effect.

### 4.8. Limitations

We confess several limitations in our study. First and foremost, we did not propose a physiological mechanism to explain the causal relationship between insomnia and tuberculosis. Secondly, the conclusions drawn based on European ancestry are not representative of the individuals of other ancestries, such as Asians and Americans, which needs to be further validated for the generalizability to non-Europeans. Additionally, since the insomnia symptom is based on self-reported information, potential recall bias and measurement error may reduce credibility to some extent. Moreover, reverse causal association of genetically proxied tuberculosis with insomnia risk cannot be analyzed due to unavailability to SNPs for tuberculosis susceptibility. Therefore, an appropriate GWAS dataset with a larger sample size is urgently warranted to clarify the reverse causal effect of tuberculosis on insomnia in the future.

## 5. Conclusion

Taken together, our bi-directional MR analysis provides evidence that there is neither any causal effect of suffering insomnia on tuberculosis susceptibility nor is there a causal effect of suffering tuberculosis on the predisposition to insomnia.

We would like to express our sincere appreciation to The Medical Research Council Integrative Epidemiology Unit at the University of Bristol Consortium and Tuberculosis Modelling and Analysis Consortium for making their GWAS summary-level statistics publicly available.

## Acknowledgments

We would like to express our sincere appreciation to MRC-IEU Consortium and Tuberculosis Modelling and Analysis Consortium for making their GWAS summary-level statistics publicly available.

## Author contributions

**Conceptualization:** Wei Zhang.

**Data curation:** Yan Feng, Jing Ge, Zhaohui Qu, Xin Li.

**Investigation:** Shiqian Wan, Zhaohui Qu, Xin Li.

**Methodology:** Shiqian Wan.

**Software:** Yan Feng, Jing Ge, Zhaohui Qu, Xin Li.

**Supervision:** Wei Zhang.

**Validation:** Shaobin Zhang, Yan Feng.

**Writing – original draft:** Shiqian Wan.

**Writing – review & editing:** Shaobin Zhang, Wei Zhang, Jing Ge, Zhaohui Qu, Xin Li.
